# An Adaptive Radon-Transform-Based Marker Detection and Localization Method for Displacement Measurements Using Unmanned Aerial Vehicles

**DOI:** 10.3390/s24061930

**Published:** 2024-03-18

**Authors:** Jianlin Liu, Wujiao Dai, Yunsheng Zhang, Lei Xing, Deyong Pan

**Affiliations:** 1 Department of Surveying Engineering & Geo-Informatics, Central South University, Changsha 410083, China; 2 School of Civil Engineering, Guangdong Construction Polytechnic, Guangzhou 510440, China

**Keywords:** Radon transform, adaptive parameters, UAV displacement measurement, marker detection

## Abstract

UAVs have been widely used in deformation monitoring because of their high availability and flexibility. However, the quality of UAV images is affected by changing attitude and surveying environments, resulting in a low monitoring accuracy. Cross-shaped markers are used to improve the accuracy of UAV monitoring due to their distinct center contrast and absence of eccentricity. However, existing methods cannot rapidly and precisely detect these markers in UAV images. To address these problems, this paper proposes an adaptive Radon-transform-based marker detection and localization method for UAV displacement measurements, focusing on two critical detection parameters, namely, the radius of marker information acquisition and the edge width of the cross-shaped scoring template. The experimental results show that the marker detection rate is 97.2% under different combinations of flight altitudes, radius ratios of marker information acquisition, and marker sizes. Furthermore, the root mean square error of detection and localization is 0.57 pixels, significantly surpassing the performance and accuracy of other methods. We also derive the critical detection radius and appropriate parameter combinations for different heights to further improve the practicality of the method.

## 1. Introduction

UAVs (unmanned aerial vehicles) are aircraft that can operate without a human pilot. They can carry out tasks through pre-programmed routes, remote control, or autonomous navigation, reducing the need for direct human intervention. UAVs are now beginning to be used to monitor and analyze deformations in civil engineering projects and infrastructure [1,2,3,4]. Their high maneuverability, easy availability and unique aerial perspective make them highly valuable. However, current UAV displacement monitoring methods have a low accuracy that is insufficient for millimeter-scale deformation monitoring. Some experiments showed that using measurement markers can significantly enhance image matching and the camera attitude estimation accuracy [5,6]. However, constantly changing imaging attitude and complex imaging environments (light, wind speed, etc.) result in blurred and noisy UAV images, which reduce the accuracy of measurement results [7]. In addition, the camera sensor’s resolution and the shadows caused by tall buildings or tree cover can also affect image interpretation and analysis. In image processing, the target coordinates cannot be accurately recognized on the map, reducing the monitoring accuracy and limiting the application of UAVs in high-precision surveys. To extend the application of UAV monitoring, it is crucial to realize high-precision and high-accuracy marker detection and localization.

UAV displacement measurement markers can be classified into three categories, inflection-point-type (“L” shaped), intersection-point-type (cross-shaped) and circular markers, which are placed within the survey area through spray painting or marker board fixing. These markers have an inflection point, intersection point and center point as the measurement points, respectively [8]. These measurement points help manual point selection on the map. Circular markers are easy to locate due to their simple graphics, but they can be affected by lighting changes and may not be accurately positioned in environments with uneven or strongly changing lighting [9]. Better than L-shaped markers, cross-shaped markers provide rich texture information and have a central symmetry, making them ideal for automatic point selection and thus improving the detection efficiency. In addition, cross-shaped markers have a clear center contrast and no eccentricity. Therefore, we chose cross-shaped markers as the measuring markers. Researchers also have designed special markers for specific applications [10,11]. However, these markers cannot be widely applied due to complicated usage conditions.

Traditionally, the coordinates of a cross-shaped marker’s measuring point are extracted manually, which is labor-intensive and inefficient. Furthermore, the accuracy of the extracted coordinates depends on the vision effect and the operator’s experience. Currently, various target detection methods are used successfully. Commonly used cross-shaped marker detection methods include the Harris algorithm [12], the template matching algorithm [13,14], and deep learning methods [15,16,17]. Cheng et al. [18] used the Harris algorithm for UAV image corner detection, enhancing the feature point extraction accuracy by using the speed-up robust feature (SURF) algorithm. This improved the quality and efficiency of UAV image matching. Azimbeik K et al. [19] designed virtual markers with special shapes and used template matching and camera calibration methods to improve image-based full-field measurements. Their method has been successfully employed to measure the displacements of a railroad bridge. With the development of deep learning algorithms in recent years, Girshick et al. [20] proposed an R-CNN algorithm, which performs a similarity analysis on the whole detected region based on the known features of the target. This allows for selection of a region with a high similarity to the sample that was input into the convolutional neural network and for detection of the target. However, deep learning methods require a lot of labeled samples for training, which is labor-intensive and leads to a low efficiency. For non-deep learning methods, the efficiency and accuracy are affected by factors such as obscured marker imaging, overexposure, sensor displacement, and noise. Xing Lei et al. [21] introduced a method based on the Radon transform principle to accurately detect and locate cross-shaped markers using saliency maps. This approach improved the robustness and accuracy of marker detection. However, when this method is used for UAV marker detection, its detection parameters are affected by the UAV flight altitude and the marker parameters, as well as the focal length and pixel size of vision sensors. This dependency prevents automated detection and may affect the detection accuracy in complex UAV route planning.

In this study, we improve the cross-shaped marker detection method based on the Radon transform by proposing an adaptive method for selecting detection parameters considering factors such as the UAV vision sensor parameters, flight altitude and marker parameters in data processing. The marker detection and localization method based on the Radon transform (referred to as the original Radon transform method) is introduced in Section 2.1. Section 2.2 presents the proposed adaptive parameter-selecting Radon transform marker detection and localization method (referred to as the adaptive Radon transform method). In Section 3, the proposed adaptive Radon transform method is compared with the traditional Harris algorithm, the template-matching method and the original Radon transform method, and the appropriate detection parameters for different flying heights are investigated. Some conclusions are drawn in Section 4.

## 2. Methods

### 2.1. Cross-Shaped Marker Detection and Localization Method Based on the Radon Transform

**Figure 1:**
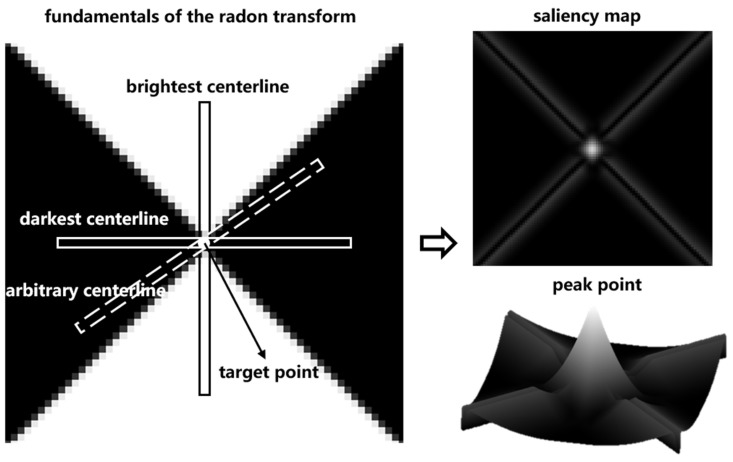
Principle of the cross-shaped marker detection and localization method based on the Radon transform.

Cross-shaped survey markers are characterized by a central symmetry. Based on this property, we define a centerline (width: 3 pixels [21]) passing through the marker center and rotate it around the center point. As Figure 1 shows, when the centerline reaches the white sector, the sum of the grayscale values inside its area is large, but when it reaches the black sector, the sum of the grayscale values inside it is small. Calculating the difference between the maximum and minimum values, we can derive the significance level of the location being the marker center. After obtaining the saliency map of the whole marker image, the sub-pixel coordinate positioning method is used to obtain the peak point of the saliency map to precisely locate the marker center.

The Radon transform $f_{R}(x,y,\theta )$ is expressed as

(1) $$f_{R}\left(x,y,\theta \right)=\sum_{i=-R}^{i=R}f(x+icos\left(\theta \right),y+isin\left(\theta \right))$$

where (x,y) denotes the target image coordinates and θ denotes the centerline rotation angle. R is the centerline radius. From Equation (1), we can calculate the sum of gray values in the centerline for any rotation angle θ.

Rotate any centerline for one rotation and calculate the square of the difference between the maximum and minimum gray values, i.e., the significance level $m_{R}(x,y)$ at point (x,y), by Equation (2).

(2) $$m_{R}\left(x,y\right)={\left(\underset{\theta_{1}\epsilon \left[0,\pi \right]}{\max}\left(f_{R}\left(x,y,\theta_{1}\right)\right)-\underset{\theta_{2}\epsilon \left[0,\pi \right]}{\min}\left(f_{R}\left(x,y,\theta_{2}\right)\right)\right)}^{2}$$

Considering both the calculation accuracy and efficiency, we here divide the centerline rotation angle into 18 groups with the same intervals, i.e., $\theta =\frac{i\pi }{18}$, i = 1, 2,…, 18.

After obtaining the saliency map, the surface fitting method is used to precisely locate the peak point to obtain the precise coordinates of the marker center.

### 2.2. Adaptive Cross-Shaped Marker Detection and Localization Method Based on the Radon Transform

The accuracy of the original Radon transform method depends on two parameters: the marker information acquisition radius R and the edge width L of the cross-shaped scoring template. The acquisition radius determines the richness of the marker image information acquired during detection. A too large radius R may contain unwanted non-marker information and leads to a huge computation load. A too small radius R may lead to insufficient marker information, affecting the detection accuracy. The edge width of the scoring template determines its graphic style, and using an unreasonable edge width can result in a low score for the true center point, causing its elimination.

**Figure 2:**
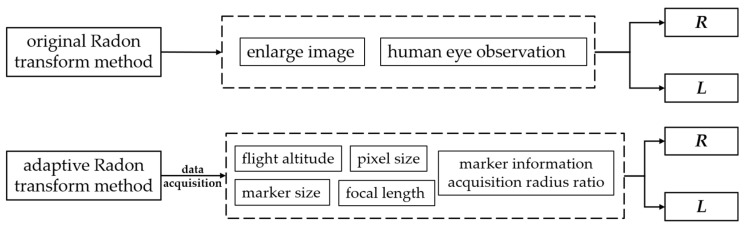
The parameter selection principles of the two methods.

At present, the original Radon transform method selects parameters using vision interpretation, which involves enlarging markers for human observations. This method has a low efficiency and is not adaptable to changing measurement conditions, leading to false detections, a low detection accuracy and undetectable markers. To solve these problems, we optimized the original Radon transform method and developed an adaptive method for selecting detection parameters (Figure 2).

#### 2.2.1. Adaptive Method for Determining the Marker Information Acquisition Radius

**Figure 3:**
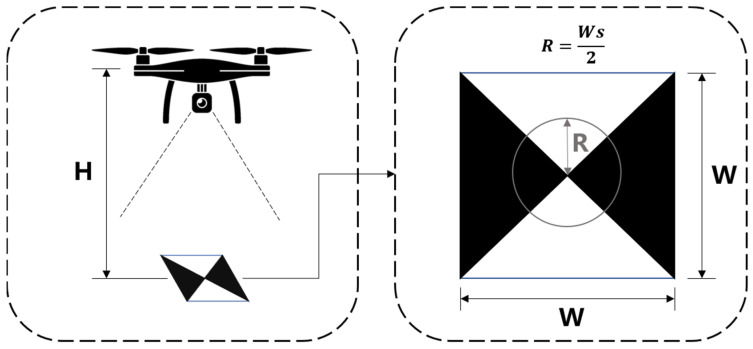
Determining the marker information acquisition radius.

During flying, the focal length f and the pixel size u of a UAV’s vision sensor remain the same. In such a case, the marker information acquisition radius is related to the flight altitude H, the marker size W∗W, and the marker information acquisition radius ratio s (Figure 3). s denotes the ratio of the marker centerline length to the side length (W), and its value range is 0<s≤1.

The ground sampling distance, GSD, is calculated by Equation (3).

(3) $$GSD=\frac{Hu}{f}$$

After obtaining the ground sampling distance, the marker information acquisition radius R can be calculated by Equation (4).

(4) $$R=\frac{W*s}{2*GSD}=\frac{Wsf}{2Hu}$$

One value of R may correspond to different combinations of {W,H,s} parameters. This paper will study the appropriate parameter combinations for different measurement conditions.

#### 2.2.2. Adaptive Method for Determining the Edge Width of a Cross-Shaped Scoring Template

**Figure 4:**
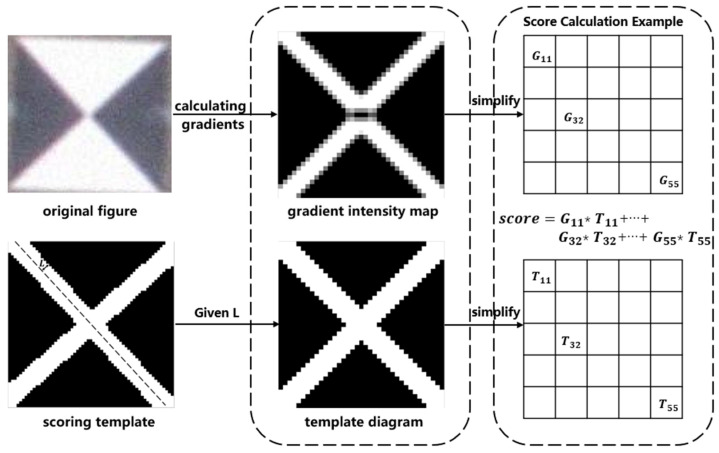
Schematic diagram of the target point scoring system. G stands for gradient intensity map and T stands for template diagram. G11 and T11 are the gray value of point (1,1) on the gradient intensity map and template diagram, respectively.

The original Radon transform method analyzes the conformity of each target point as a centroid using a scoring system. A higher score indicates a higher conformity. The width of the cross-shaped pattern in the scoring template, determined by the edge width *L*, plays a crucial role in the scoring calculation, as depicted in Figure 4. A larger or smaller width of the cross-shaped pattern leads to a reduced accuracy in marker detection and localization. Therefore, using a well-designed scoring template can improve the accuracy of marker detection.

**Figure 5:**
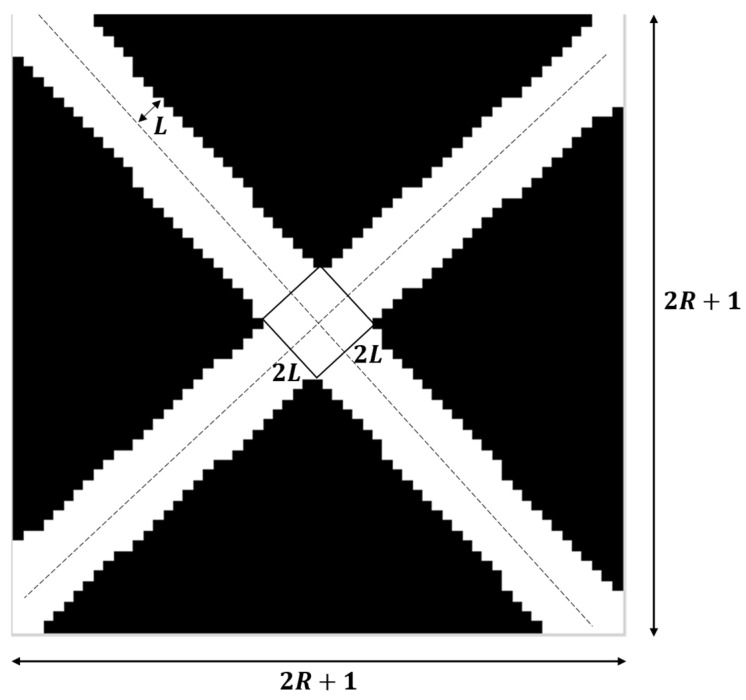
Selecting the edge width of a cross-shaped scoring template.

When normalizing the gradient intensity map, we define pixel values greater than 0 as bright pixels while the rest are dark pixels. In a gradient intensity map, if bright pixels are predominantly found at the junction between black and white regions, the target point has a high possibility of being the marker center. The number of bright pixels k is counted and is taken as the theoretical sum of bright pixels in the template map. If the bright pixels are evenly distributed at the junction of black and white areas, we can calculate the edge width L of the template bright pixels as in Figure 5.

Firstly, the diagonal length D of the template is calculated via Equation (5).

(5) $$D=\sqrt{2}(2R+1)$$

According to the principle that the number of theoretical bright pixels is approximately equal to the number of statistical bright pixels (k), Equation (6) is obtained.

(6) $$2(2L)D-{\left(2L\right)}^{2}=k$$

Then, the edge width of the template is obtained using

(7) $$L=\frac{\left(D-\sqrt{D^{2}-k}\right)}{2}$$

## 3. Experiments and Analysis

**Figure 6:**
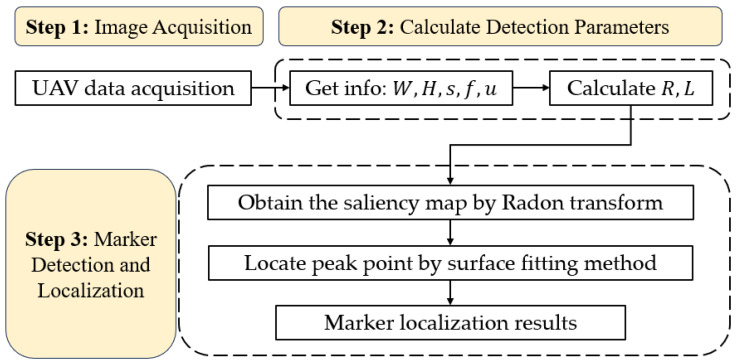
Flowchart of the proposed adaptive method based on the Radon transform.

This section verifies the adaptive methods for parameter selection introduced in Section 2.2.1 and Section 2.2.2. It also investigates the appropriate parameter combinations for different measurement conditions. The adaptive method is compared with other marker detection methods. The flowchart of the proposed adaptive method in this paper is shown in Figure 6.

### 3.1. Critical Detection Radius

The critical detection radius $R_{t}$ refers to the minimum radius in which the marker center point can be detected. Only when $R_{t}<R<R_{m}$ is satisfied ($R_{m}$ being the pixel length of the marker radius) can optimal marker detection be achieved. Obtaining the value of $R_{t}$ can exclude a large number of {W,H,s} parameter combinations, reducing the workload involved. $R_{t}$ is calculated as follows: any two parameters in the combination of {W,H,s} parameters are chosen, and only one parameter is adjusted downwards from its optimal value. If the marker cannot be detected, the R value of the previous detected set of markers is taken as a candidate for $R_{t}$. After all the data have been processed, the largest $R_{t}$ candidate is the final $R_{t}$.

The UAV utilized in the experiment is the DJI Phantom 4 RTK. The weather during the experiment was sunny (23 °C) with a breeze. The UAV camera parameters are shown in Table 1.

**Figure 7:**
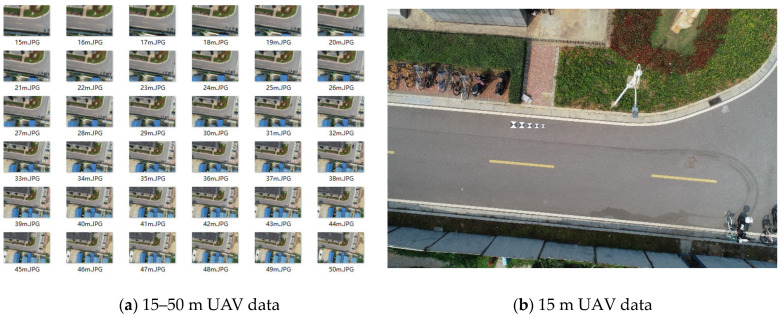
Experimental data.

As shown in Figure 7, data collection and processing were designed as follows:

(1) H: images were acquired at different heights from 15 m to 50 m with a step size of 1 m;
(2) W: five targets of {20, 25, 30, 35, 40} cm were laid at each height;
(3) s: each target was processed with six presets ratios {1/4, 1/3, 1/2, 2/3, 3/4, 1}.

The R value of different parameter combinations was calculated via Equation (4) and the results are shown in Table 2.

The maximum R value was determined via Equation (8) and taken as the critical radius $R_{t}$.

(8) $$R_{t}=\underset{i\in [0,15]}{\max}R_{i}$$

where $R_{i}$ is the R of each parameter combination. The final critical detection radius is 6.

### 3.2. Appropriate Combinations of Detection Parameters

**Figure 8:**
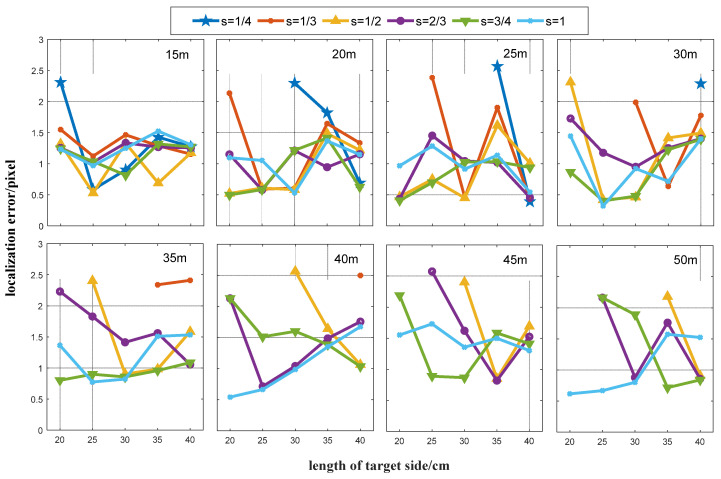
Detection accuracy for different {W, H, s} parameter combinations. Flight heights are recorded in the right upper corner of each figure.

The R value calculated by Equation (4) is affected by the parameters W, H, and s. When determining an R value, we should consider factors like the marker installation difficulty, image quality, and marker detection calculation efficiency. Therefore, W and s should be minimized while maintaining the detection accuracy. Since UAV measurements have specific flight altitude requirements, the value of H fluctuates around a constant value. In this study, the detection accuracy is measured by the distance between the detected target point and the manually selected target point. The manually selected target point locations were obtained by averaging at least three selected points, and any R values smaller than $R_{t}$ were rounded off during processing. The same experimental data as in Section 3.1 were used for this experiment. We started from a height of 15 m and a step size of 5 m. The results are shown in Figure 8.

As shown in Figure 8, when the flight height is low, all the five target sizes are able to detect the marker center at all six acquisition radius ratios. As the height increases, the combination of a small target size and a small radius ratio appears to be unable to detect the marker ($R<R_{t}$). Additionally, as the value of R gets closer to $R_{t}$ ($R>R_{t}$), the accuracy of marker detection decreases. At the flight altitude of 50 m, all targets are only detected at s=1. We analyzed the results as follows:

(1) The minimum detection error $e_{h}$ was determined for each height of $h\in \left\{15,20\ldots 45,50\right\}\;m$;
(2) The accuracy threshold τ was set and all detection errors e were found that satisfy $e_{h}\leq e\leq e_{h}+\tau$ at height h;
(3) As the value of s increases, the difficulty of marker installation is more significant than the improvement in the computational efficiency. In this paper, we give priority to W rather than s. Therefore, among all the detection errors that satisfy the conditions in (2), we prioritized the combination of parameters with a small marker size as the appropriate detection parameter.

The analysis results are shown in Table 3.

The errors were calculated by the following equations:

(9) $$Avg\_RMSE=(e_{1}+e_{2}+\ldots +e_{i})/i$$

(10) $$Min\_Error=\underset{i}{\min}e_{i}$$

where Avg_RMSE denotes the RMSE of all parameter combinations, i denotes the number of parameter combinations that can detect the marker, Min_Error denotes the minimum detection error at this height, and Error denotes the detection error of the best parameter combination in this paper. We have identified appropriate parameter combinations at various heights and will use them in the following sections.

### 3.3. Performance of the Adaptive Radon Transform Method

**Figure 9:**
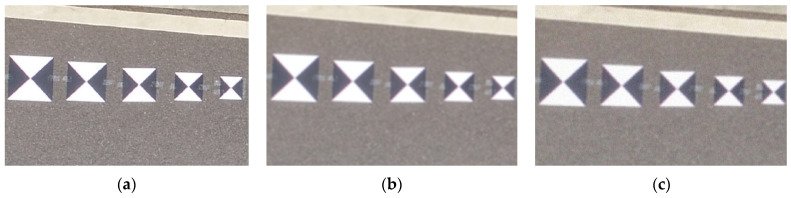
(**a**) Original image, (**b**) fuzzy image, (**c**) fuzzy and noisy image.

In this section, the performance of the proposed adaptive Radon transform method is assessed by comparing it with the original Radon transform method, the template matching method, and the Harris corner point detection method separately. For the experiment, we used the data in Section 3.1 after Gaussian blurring and adding Gaussian noise to simulate the low image quality that occurs during real UAV operations (Figure 9).

**Figure 10:**
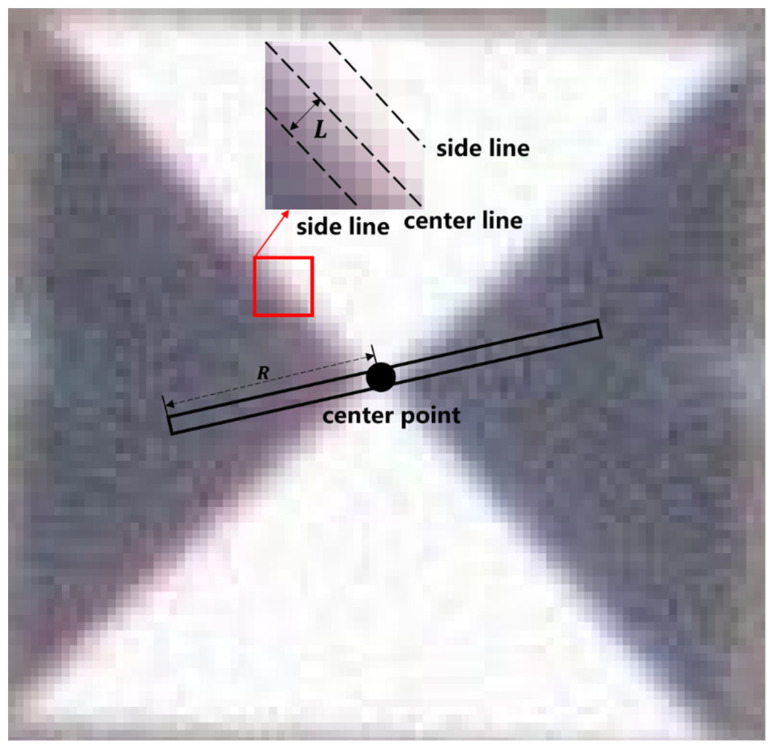
Manually selected parameters for the original Radon transform method.

For the proposed method, the parameter combinations for different flying heights were selected according to the results of Section 3.2 using the nearest-neighbor rule. For example, when H = {18, 19, 20, 21, 22} m, all parameter combinations were selected based on H = 20 m. The original Radon transform method selects parameters based on vision experience (Figure 10). The marker information acquisition radius was determined by human judgment, and the pixel length of the marker image at that radius was acquired through statistical analysis. The edge width of the cross-shaped scoring template was determined by the distance between the edge and the centerline of the black and white areas. In this experiment, the selected heights, R and L are 15–50 m, 11 pixels, and 1.6 pixels, respectively. The same parameters were used in the Harris algorithm as those in paper [12]. The template matching method determines a square template that can be uniformly divided into four rectangles. Two rectangles are defined as black sections and two others are white sections. These two colored sections are interlaced. The marker center is located by matching the square template with the image.

We used the four methods to detect the markers in low-quality images at all heights. If the distance between the detected marker and the manually selected marker is greater than 3 pixels, detection is considered to have failed. The results are shown in Table 4.

**Figure 11:**
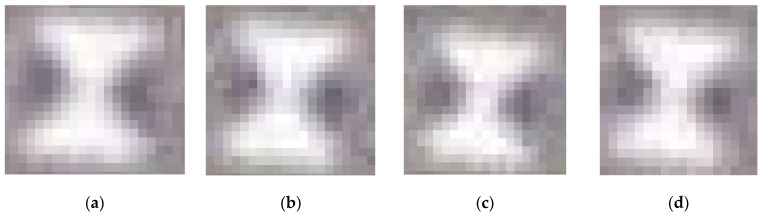
Failed image detection using this paper’s method: (**a**) H = 47 m and W = 20 cm; (**b**) H = 48 m and W = 20 cm; (**c**) H = 49 m and W = 20 cm (**d**) H = 50 m and W = 20 cm.

As shown in Table 4, the accuracy of the proposed method is much higher than that of the template matching method and the Harris corner point detection method and is also higher than the original Radon-transform-based detection method. The proposed method failed at detecting markers with a size of 20 cm at flight altitudes of 47, 48, 49, and 50 m (Figure 11).

The proposed method failed at detecting markers with a size of 20 cm at flight altitudes of 47, 48, 49, and 50 m (Figure 11). These images have some similarities: the marker’s radius is under 6 pixels, the black sector area is severely lacking, and the center area is blurred. These features limit marker detection. At flight altitudes of 47 m or above, the marker radius should be larger than 20 cm for detection.

**Figure 12:**
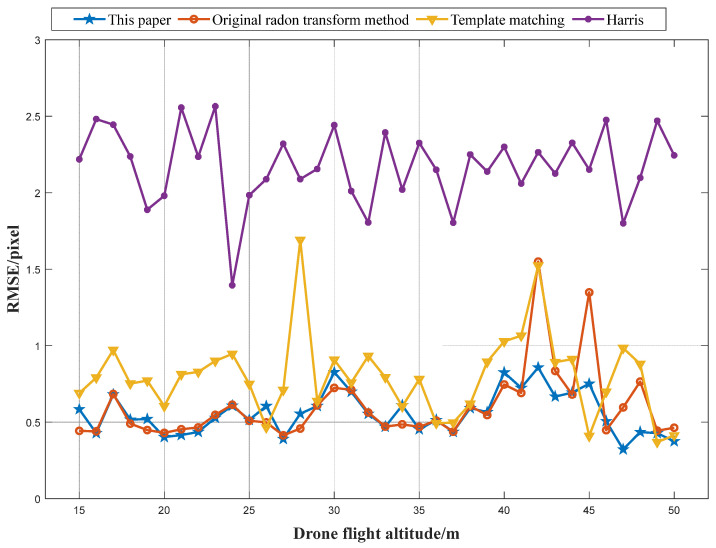
RMSE of the four methods for five markers at different heights.

After detecting markers, we performed marker localization using a surface-fitting method [22]. The Radon transform method locates markers in the saliency map, whereas other methods locate markers in the original image. To evaluate accuracy, we calculated the RMSE of the five markers at each height for all four methods. The results are shown in Figure 12.

The results in Figure 12 show that the proposed method outperforms the other three methods in terms of the marker localization accuracy, which is as high as within 1 pixel. The overall accuracy of the original Radon transform method is high, but the positioning can be inaccurate at certain heights. This is because parameter selection is influenced by operator experience and the complex and changing image situation. The selected parameters may not be adaptable to the image data, resulting in a low accuracy. The proposed method has a higher accuracy than the template matching method and the Harris detection method, primarily because the latter two methods rely heavily on the original image quality, which is often compromised in UAV photography. The proposed adaptive Radon transform method locates markers in the saliency map. The original image quality only affects the circular spot size in the saliency map, but also has no effect on the location of the peak point. Therefore, using the generated marker saliency map for marker localization leads to a high accuracy.

As shown in Table 5, the proposed method outperforms the template matching method and Harris algorithm with regard to the detection success rate and localization accuracy. Additionally, compared with the original Radon transform method, the proposed method avoids many manual operations, greatly improving the efficiency and also improving the detection success rate and localization accuracy. In complex imaging environments, the proposed method is able to achieve a balance between efficiency and accuracy. It ultimately contributes to the achievement of a high precision, a high efficiency and automation of UAV displacement measurements.

### 3.4. Displacement Measurement Experiment

**Figure 13:**
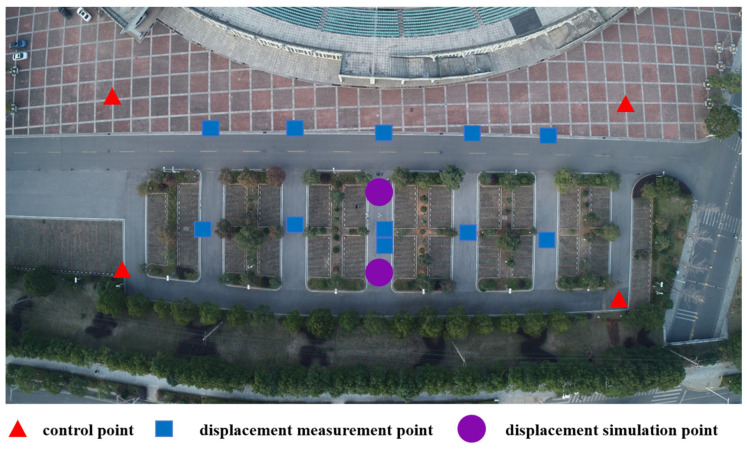
Layout of the measurement markers at the experimental site.

**Figure 14:**
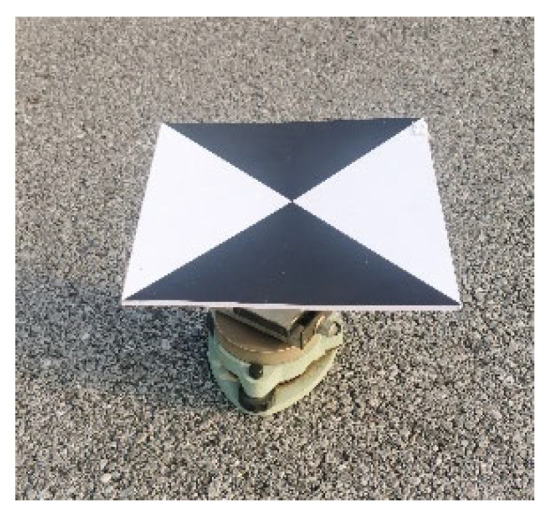
Three-dimensional slide table displacement simulation point.

In order to evaluate the effectiveness of the method proposed in this paper for dis-placement measurements, we carried out three-dimensional displacement measurement experiments in an area of about 10,000 square meters located directly south of Central South University’s stadium. The specific experimental flow is as follows:

(1) Lay out measurement markers and acquire UAV images in four missions. The measurement markers were laid out as shown in Figure 13. Four marker control points, eleven marker displacement measurement points and two 3D slide table displacement simulation points were laid out. The markers were set at a size of 20 ∗ 20 cm based on the results in Section 3.2. The main body of the 3D simulation point is a three-axis slide unit (with an accuracy of 1 mm) with a measurement mark fixed on the top. The slide scale can be adjusted to set the true displacement value (Figure 14). Control points were measured using a Leica TS09 model (Leica, Wetzlar, Germany) total station with an accuracy of 2.2 mm.

(2) Select the data of the first mission and locate all measurement markers using the proposed method, the original Radon transform method, the Harris algorithm, and the template matching method separately. Reconstruct the 3D model.

(3) Export the 3D coordinates of the displacement simulation points. Select the data of the second mission and repeat steps (1) and (2) to obtain the 3D coordinates of the displacement simulation points.

(4) Analyze the difference in the 3D coordinates of the displacement simulated points obtained from the two missions. Then, we can obtain the displacement measurements of these two UAV missions via Equation (11).

(11) $$D_{ij}^{n}=\sqrt{{\left(x_{nj}-x_{ni}\right)}^{2}+{\left(y_{nj}-y_{ni}\right)}^{2}+{\left(z_{nj}-z_{ni}\right)}^{2}}$$

where $\left(x_{ni},y_{ni},z_{ni}\right)$ and $\left(x_{nj},y_{nj},z_{nj}\right)$ denote the three-dimensional coordinates of the nth measurement point computed during the ith and jth UAV missions, respectively, and $D_{ij}^{n}$ denotes the displacement measurement result of the nth measurement point in two adjacent UAV missions.

The weather during the experiment was sunny with a breeze. The other conditions during the experiment are shown in Table 6.

The final displacement results of the displacement simulation points were obtained as shown in Table 7.

Based on the results in Table 7, the RMSE of the displacement measurements of the four methods was calculated and is shown in Table 8.

As the displacement measurement accuracy in Table 8 shows, the proposed method outperforms the original Radon transform method, template matching method and Harris algorithm. This validates the effectiveness of the proposed method for 3D displacement deformation measurements.

## 4. Conclusions

This paper introduces an adaptive marker detection and localization method based on the Radon transform for solving problems in UAV displacement measurements, such as the low efficiency, precision and automation in detecting markers from low-quality images. We first study the principle and method of the original marker detection method based on the Radon transform, analyzing its limitations in UAV displacement measurements. By focusing on two key detection parameters, namely, the marker information acquisition radius and the cross-shaped scoring template edge width, we propose an adaptive marker detection method based on the Radon transform specifically applicable to marker detection in UAV measurement images.

The experimental results demonstrate that the proposed method can automatically derive the necessary measurement parameters at different flight altitudes. This greatly reduces the manual marker selection time and enhances accuracy and practicality compared with the original Radon transform method. The proposed method exhibits a higher detection success rate and accuracy and stronger noise and ambiguity resistance under complex imaging conditions compared to other methods. In the displacement measurement experiments, the proposed method displays a higher displacement measurement accuracy than the original Radon transform method, the template matching method and the Harris algorithm, demonstrating its practicality for displacement measurements. The experiments in this paper were conducted in realistic settings using standard equipment and materials, ensuring the universal applicability of the results.

This work also has some limitations. The parameter combinations obtained in Section 3.2 may not be optimal for all actual engineering applications involving a wider range of flight altitudes and different marker installation conditions. Therefore, parameter combinations should be refined in subsequent engineering applications.

## Figures and Tables

**Table 1:** UAV camera parameters.

Parameter Name	Parameter Data
type	FC6310R
resolution	5472 × 3648
focal length	8.8 (mm)
pixel size	2.4 (um)

**Table 2:** R value of different parameter combinations.

	W (cm)	s	H (m)	R (Pixel)
Group 1	20	1/4	18	5
Group 2	20	1/3	23	5
Group 3	20	1/2	34	5
Group 4	20	2/3	46	5
Group 5	20	3/4	46	5
Group 6	25	1/4	21	5
Group 7	25	1/3	27	5
Group 8	25	1/2	34	6
Group 9	30	1/4	21	6
Group 10	30	1/3	37	4
Group 11	30	1/2	46	5
Group 12	35	1/4	28	5
Group 13	35	1/3	39	5
Group 14	40	1/4	32	5
Group 15	40	1/3	42	5

**Table 3:** Appropriate detection and localization parameters and accuracy at different flying heights.

Flying Height	W/cm	s	Avg_RMSE */Pixel	Min_Error/Pixel	Error/Pixel
15 m	25	1/2	1.25	0.53	0.53
20 m	20	3/4	1.18	0.50	0.50
25 m	20	3/4	1.15	0.39	0.41
30 m	25	1	1.32	0.32	0.32
35 m	25	1	1.50	0.77	0.77
40 m	20	1	1.57	0.54	0.54
45 m	25	3/4	1.60	0.82	0.89
50 m	20	1	1.43	0.62	0.62

* RMSE: root mean square error.

**Table 4:** Marker detection results of different methods.

Methods	Number of Correct Detections	Total Number of Detections	Correct Detection Rate (%)
Proposed method	176	180	97.8
Original Radon transform method	171	180	95.0
Template matching method	124	180	68.9
Harris algorithm	133	180	73.9

**Table 5:** Performance of different methods.

	Detection Rate/(%)	RMSE /(Pixel)	Time for One Set of Data /(s)
Proposed method	97.8	0.57	1
Original Radon transform method	95.0	0.64	10
Template matching method	68.9	0.84	1
Harris algorithm	73.9	2.19	0

**Table 6:** Experimental conditions.

Condition	
flying height	25 (m)
flying speed	2.4 (m/s)
GSD	0.68 (cm/pixel)
overlap rate (vertical/horizontal)	80%/75%
camera tilt angle	−60°

**Table 7:** Three-dimensional slide table displacement simulation point measurement results.

	Displacement Simulation Point 1			Displacement Simulation Point 2		
	Group 1	Group 2	Group 3	Group 1	Group 2	Group 3
Preset displacement (mm)	11.5	16.7	10.0	5.0	10.0	15.0
Proposed method (mm)	15.3	18.7	8.5	9.9	13.6	15.4
Original Radon transform method (mm)	17.9	12.6	7.9	12.0	18.1	16.3
Template matching method (mm)	23.3	22.7	7.0	18.7	16.0	9.6
Harris algorithm (mm)	28.0	24.1	25.7	34.9	20.3	29.8

**Table 8:** Displacement measurement RMSE values of different methods.

Methods	RMSE (mm)
Proposed method	3.1
Original Radon transform method	5.5
Template matching method	8.5
Harris algorithm	17.3

## Data Availability

Data are contained within the article.
